# Extreme temperature increases the risk of stillbirth in the third trimester of pregnancy

**DOI:** 10.1038/s41598-022-23155-3

**Published:** 2022-11-02

**Authors:** Hsiao-Yu Yang, Jason Kai Wei Lee, Chia-Pin Chio

**Affiliations:** 1 Institute of Environmental and Occupational Health Sciences, National Taiwan University College of Public Health, No. 17 Xuzhou Road, Taipei, 10055 Taiwan; 2 Department of Public Health, National Taiwan University College of Public Health, Taipei, Taiwan; 3 Department of Environmental and Occupational Medicine, National Taiwan University Hospital, Taipei, Taiwan; 4 Human Potential Translational Research Programme, Yong Loo Lin School of Medicine, National University of Singapore, Singapore, Singapore; 5 Department of Physiology, Yong Loo Lin School of Medicine, National University of Singapore, Singapore, Singapore; 6 Global Asia Institute, National University of Singapore, Singapore, Singapore; 7 N.1 Institute for Health, National University of Singapore, Singapore, Singapore; 8 Institute for Digital Medicine, National University of Singapore, Singapore, Singapore; 9 Singapore Institute for Clinical Sciences, Agency for Science, Technology, and Research (A*STAR), Singapore, Singapore; 10 Department of Medical Research, Tung’ Taichung Metro Harbor Hospital, Taichung, Taiwan

**Keywords:** Reproductive disorders, Climate change

## Abstract

Epidemiological studies have reported the association between extreme temperatures and adverse reproductive effects. However, the susceptible period of exposure during pregnancy remains unclear. This study aimed to assess the impact of extreme temperature on the stillbirth rate. We performed a time-series analysis to explore the associations between temperature and stillbirth with a distributed lag nonlinear model. A total of 22,769 stillbirths in Taiwan between 2009 and 2018 were enrolled. The mean stillbirth rate was 11.3 ± 1.4 per 1000 births. The relative risk of stillbirth due to exposure to extreme heat temperature (> 29 °C) was 1.18 (95% CI 1.11, 1.25). Pregnant women in the third trimester were most susceptible to the effects of extreme cold and heat temperatures. At lag of 0–3 months, the cumulative relative risk (CRR) of stillbirth for exposure to extreme heat temperature (29.8 °C, 97.5th percentile of temperature) relative to the optimal temperature (21 °C) was 2.49 (95% CI: 1.24, 5.03), and the CRR of stillbirth for exposure to extreme low temperature (16.5 °C, 1st percentile) was 1.29 (95% CI: 0.93, 1.80). The stillbirth rate in Taiwan is on the rise. Our findings inform public health interventions to manage the health impacts of climate change.

## Introduction

Global warming is a critical public health emergency. It has increased the global temperature average by approximately 0.5 °C over the past 50 years, and the frequency and severity of extreme temperatures^1^. Non-optimal temperature exposure is associated with a substantial mortality burden, accounting for 9.43% of all deaths; among them, 8.52% were cold-related, and 0.91% were heat-related^2^. There is increasing evidence that ambient temperature influences adverse birth outcomes^3^. The stillbirth rates had regional differences^4^.

The UN Inter-Agency Group for Child Mortality Estimation (UN-IGME) reported nearly two million stillbirths occurred in 2019, with most stillbirths occurring in sub-Saharan Africa and south Asia^4^. Taiwan is a subtropical island country in East Asia; it has a humid climate and is susceptible to global warming. In Taiwan, hot seasons are long, lasting from May to September. The winter is short and mild, with there being no severe cold temperature in the winter. The mean annual high temperature on the island is 21 °C (70 ºF) (https://www.britannica.com/place/Taiwan/Climate). From 1911 to 2005, Taiwan's temperature warmed by 1.4 °C, indicating that warming in Taiwan is occurring approximately twice as fast as that in the Northern Hemisphere (0.7 °C)^5^. Taiwan is suitable for observing the impact of global warming on stillbirths.

This study aimed to examine the effects of extreme temperature on the stillbirth rate in Taiwan. First, we determined associations between ambient temperature and stillbirth risk. Then, we estimated the number of stillbirths attributable to hot and cold temperatures in Taiwan and explored the trends of stillbirths.

## Results

**Figure 1:**
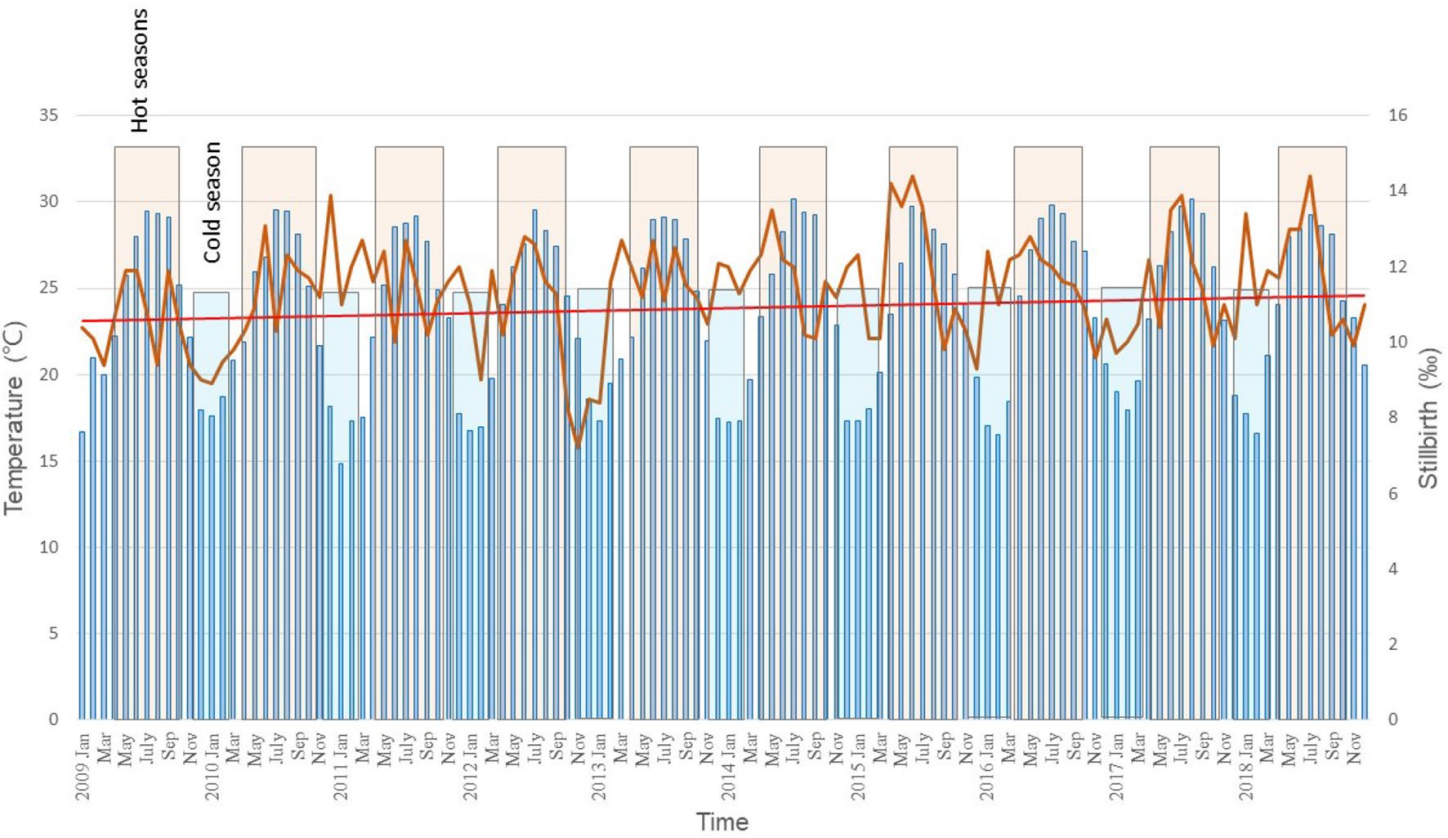
Monthly average temperatures and stillbirth rates in Taiwan from January 2009 to December 2018. The monthly average temperatures (blue bar charts) show a seasonal pattern. There was an increased stillbirth rate (orange line) in the hot seasons. The red line is the trendline of stillbirths, showing an increased risk of stillbirth in Taiwan over time.

**Figure 2:**
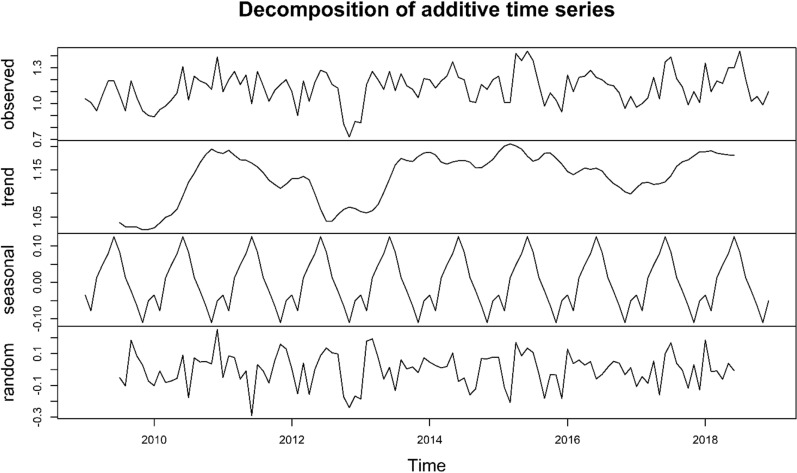
Decomposition of time-series data of the stillbirth rate in Taiwan from January 2009 to December 2018. A seasonal time series consists of a trend component, a seasonal component, and an irregular component. Seasonality has a bimodal distribution with a primary peak in hot seasons and a minor peak in cold seasons. There is an increasing trend of the stillbirth rate in Taiwan.

A total of 22,769 stillbirths occurred in Taiwan from January 1, 2009, to December 31, 2018. The mean stillbirth rate was 11.3 (SD 1.4) per 1000 births. The stillbirth rate was highest in June, at 12.6 (SD 1.2) per 1000 births. The mean monthly temperature and relative humidity were 23.9 °C (median 24.4, range 14.8–30.2) and 75% (median 75, range 66–82), respectively (Supplementary Table 1). There was a seasonal stillbirth pattern, with a primary peak in hot seasons and a minor peak in cold seasons (Fig. 1). When we decomposed the time series data of stillbirths in Taiwan, we observed that stillbirths had seasonality that occurred over a fixed period. Seasonality has a bimodal distribution with a primary peak in hot seasons and a minor peak in cold seasons. There is an increasing trend of the stillbirth rate in Taiwan (Fig. 2). Though there was a positive association between temperature and the stillbirth rate (Pearson correlation coefficient = 0.30, *p*-value = 0.01), the association between temperature and the stillbirth rate was nonlinear. The minimum stillbirth rates were observed at temperatures between 20 and 22 °C (Supplementary Fig. 1). The relative risks (RRs) of stillbirth due to exposure to cold (< 20 °C), mild heat (22–25 °C), moderate heat (25–29 °C), and extreme heat (> 29 °C) were 1.08 (95% CI 1.02, 1.14), 1.10 (95% CI 1.04, 1.17), 1.15 (95% CI 1.09, 1.22), and 1.18 (95% CI 1.11, 1.25), respectively. Non-optimal temperature (either cold or heat) exposure was responsible for 10.24% (95% CI 0.06, 0.15) of stillbirths; among them, hot temperature exposure was responsible for a higher attributable fraction (AF) of stillbirths (8.34%, 95% CI 0.05, 0.11) than cold temperature exposure (1.89%, 95% CI 0.01, 0.03). Extreme heat exposure (> 29 °C) was responsible for 2.64% (0.02, 0.03) of the total stillbirths (Table 1).

**Table 1:** Relative risk and attributable fraction of stillbirth related to mild, moderate, and extreme heat.

	Total births (No.)	Stillbirths (No.)	Adjusted RR (95% CI)^a^	Attributable fractions (%) (95% CI)	Attributable stillbirths (95% CI)
Cold (< 20 °C)	555,504	6053	1.08 (1.02, 1.14)**	7.11% (0.02, 0.12)	431 (120, 728)
Optimum (20–22 °C)	157,088	1699	Referent	Referent	–
Mild heat (22–25 °C)	365,724	3935	1.10 (1.04, 1.17)***	9.18% (0.04, 0.14)	361 (152, 560)
Moderate heat (25–29 °C)	604,404	7079	1.15 (1.09, 1.22)***	13.69% (0.05, 0.22)	936 (599, 1259)
Extreme heat (> 29 °C)	336,837	3999	1.18 (1.11, 1.25)***	15.06% (0.10, 0.20)	602 (404, 790)

RR, relative risk; CI, confidence interval.

* *p*-value < 0.05, ** *p*-value < 0.01, *** *p*-value < 0.001.

^a^Adjusted for relative humidity. The Akaike information criterion (AIC) for the model was 1262.

**Figure 3:**
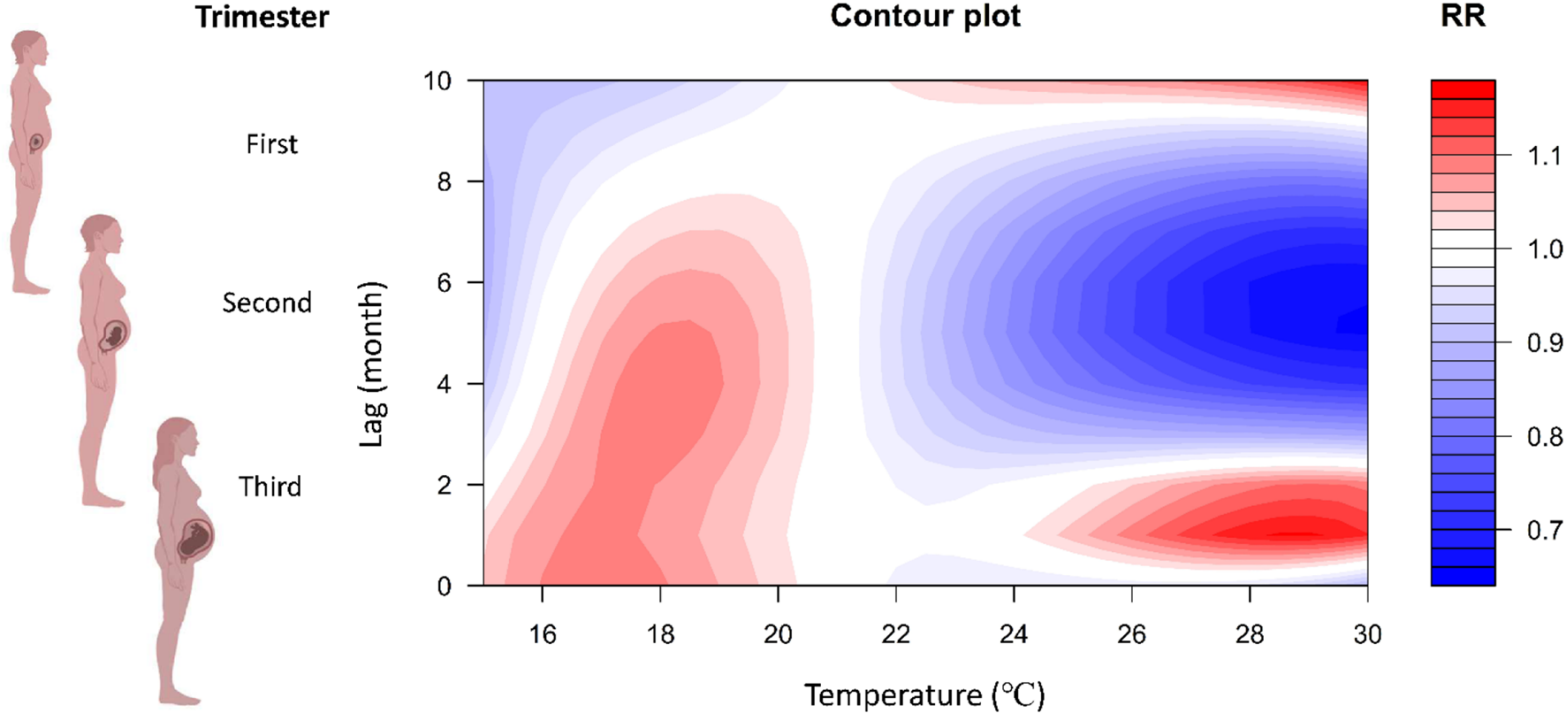
Contour plots of the thermal effect of time lags and temperature on the RR of stillbirth, with the reference of 21 °C. Taiwan, 2009–2018. This figure shows that exposure to both hot and cold temperatures increases the risk of stillbirth in pregnant women in the second and third trimesters. The figures of pregnant women were created with BioRender.com.

**Figure 4:**
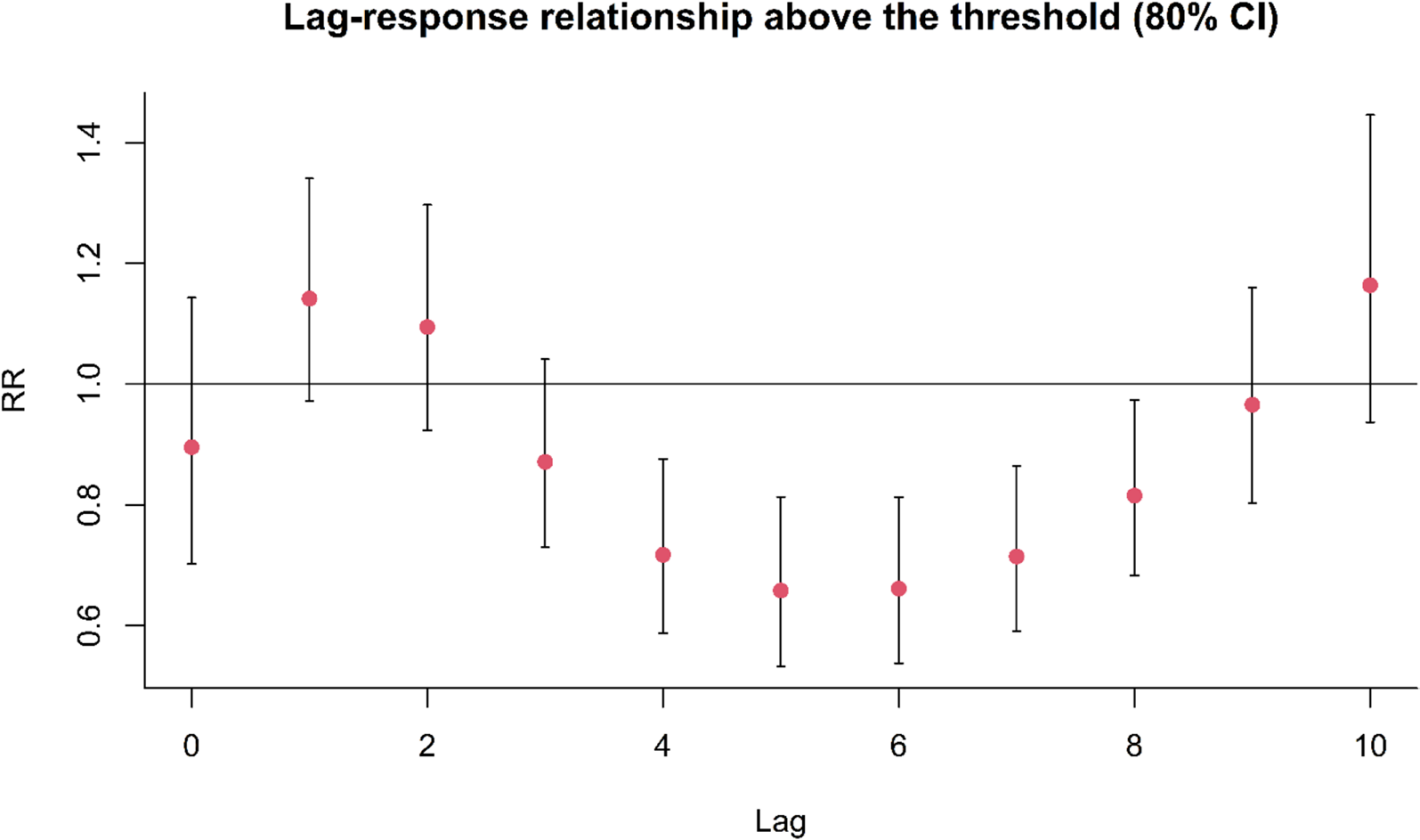
The association between temperature and stillbirth summarized by the RR of stillbirth associated with exposure to extreme heat temperatures (29.8 °C, the 97.5th percentile) above the optimal temperature (21 °C). The lag response curve shows the existence of the mortality displacement for extreme heat temperature. The effects of extreme heatt temperatures on stillbirth was largest at lag 1 month and then reduced below baseline from lag 3 to 8 months.

**Figure 5:**
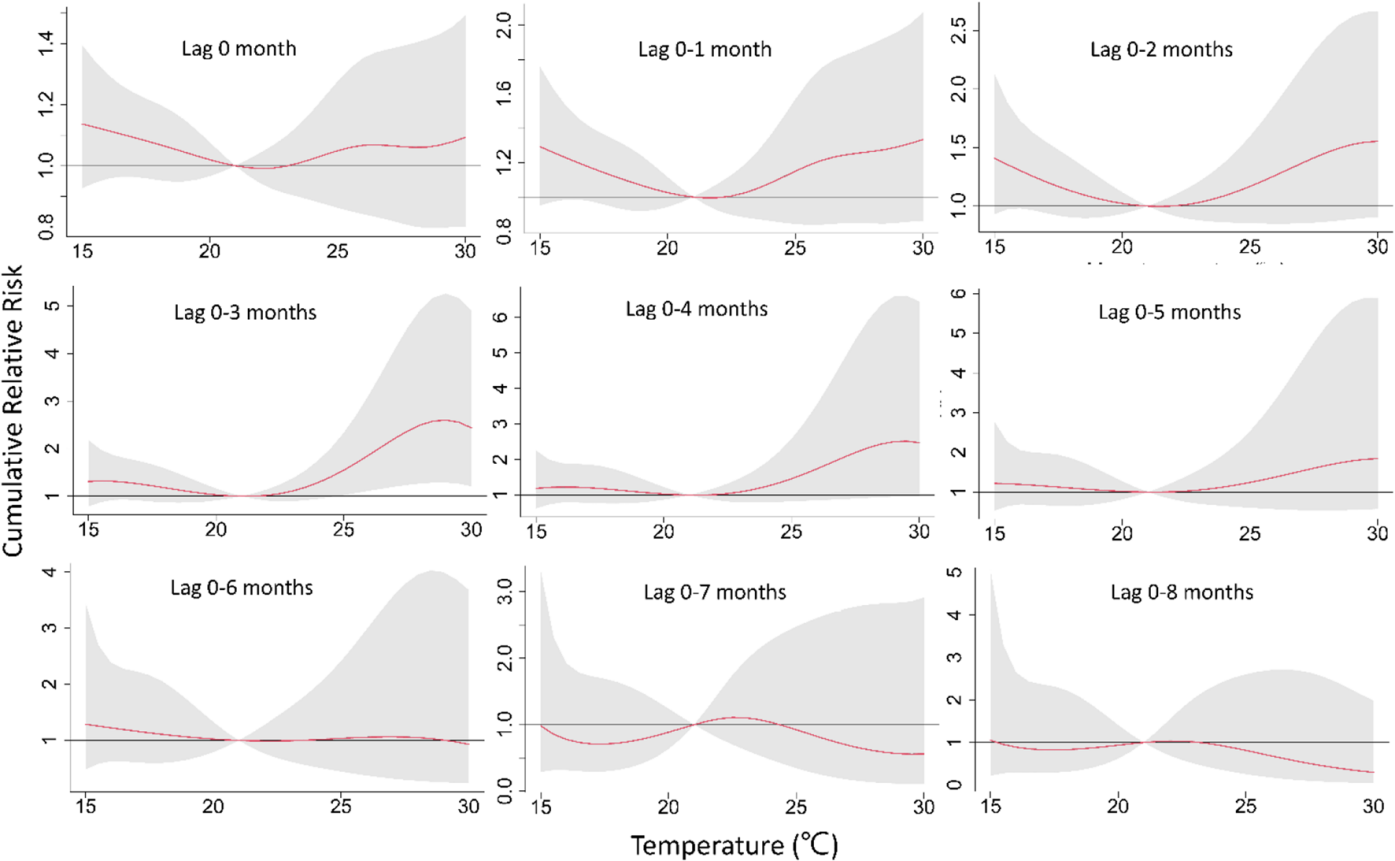
Exposure–response curves of monthly mean temperatures and cumulative RRs of stillbirth using quasi-Poisson regression with the DLNM and different lag periods; the reference corresponds to 21 °C. Taiwan, 2009–2018. Cumulative RRs (red line) and 95% CIs (gray zone) for stillbirths over different lag periods (0, 0–1, 0–2, 0–3, 0–4, 0–5, 0–6, 0–7, 0–8, and 0–9 months). Estimates are relative to the optimal temperature (21 °C) for the study area as a reference. We observed a U-shaped association between stillbirth risk and temperature during lag 0–1, 0–2, and 0–3 months. Heat stress has the highest cumulative RR during the third trimester (lag 0–3 months). The overall cumulative effect of hot temperatures was larger than that of cold temperatures.

An overall picture of the effect of temperature on stillbirth using a contour map of the RR with temperatures and lag times was compared with the reference value at 21 °C, which represents the minimum stillbirth rate. The plot shows a very strong and immediate effect of heat at lag 0–2 months before delivery and suggests a more delayed effect of cold at lag 0–6 months. In the third trimester (lag 0–4 months), extreme hot and cold temperatures increased the risks of stillbirth (Fig. 3). Figure 4 shows a clear harvesting effect of extreme heat on stillbirth, and the heat stress had the greatest impact on stillbirth one month before delivery (lag 1 month). The associations between stillbirth and temperature were U-shaped at lag 0–4 months, indicating that pregnant women were more sensitive to the influence of temperature during the third trimester of pregnancy. The overall cumulative effect of hot temperatures was more significant than cold temperatures (Fig. 5). The greatest cumulative effect of extreme heat temperature was found in the 97.5th percentile of temperature (29.8 °C) relative to the optimal temperature (21 °C) at lag 0–3 months, with a cumulative relative risk (CRR) of 2.49 (95% CI: 1.24, 5.03). The largest CRR of stillbirth in those exposed to extreme low temperatures was observed for the 1st percentile of temperature (16.5 °C) relative to the optimal temperature (21 °C), with a CRR of 1.29 (95% CI: 0.93, 1.80) at lag 0–3 months (Table 2).

**Table 2:** The cumulative relative risk (CRR) of stillbirth in extreme heat temperature (99th and 97.5th percentile) and extreme cold temperature (1st and 2.5th percentile) relative to the optimal temperature (21 °C).

Lag effect	99th percentile (30.1 °C) relative to 21 °C, CRR (95% CI)	97.5th percentile (29.8 °C) relative to 21 °C, CRR (95% CI)	2.5th percentile (16.7 °C) relative to 21 °C, CRR (95% CI)	1st percentile (16.5 °C) relative to 21 °C, CRR (95% CI)
Lag 0	1.10 (0.80, 1.50)	1.09 (0.80, 1.48)	1.10 (0.96, 1.25)	1.10 (0.96, 1.27)
Lag 0–1	1.34 (0.86, 2.09)	1.33 (0.86, 2.05)	1.19 (0.98, 1.43)	1.20 (0.99, 1.46)
Lag 0–2	1.55 (0.91, 2.67)	1.55 (0.90, 2.67)	1.24 (0.96, 1.60)	1.25 (0.97, 1.63)
Lag 0–3	2.40 (1.19, 4.8)*	2.49 (1.24, 5.03)*	1.28 (0.92, 1.77)	1.29 (0.93, 1.80)
Lag 0–4	2.46 (0.95, 6.40)	2.50 (0.96, 6.53)	1.22 (0.79, 1.87)	1.22 (0.80, 1.88)
Lag 0–5	1.85 (0.58, 5.89)	1.84 (0.57, 5.91)	1.17 (0.69, 2.00)	1.17 (0.69, 2.00)
Lag 0–6	0.92 (0.23, 3.64)	0.95 (0.24, 3.76)	1.18 (0.62, 2.25)	1.19 (0.63, 2.27)
Lag 0–7	0.56 (0.11, 2.94)	0.56 (0.11, 2.88)	0.72 (0.30, 1.75)	0.73 (0.30, 1.78)
Lag 0–8	0.29 (0.04,1.96)	0.31 (0.05,2.04)	0.84 (0.29, 2.40)	0.85 (0.30, 2.43)
Lag 0–9	0.36 (0.04, 3.67)	0.38 (0.04, 3.75)	0.80 (0.21,3.10)	0.76 (0.19, 3.00)

*p < 0.05.

**Figure 6:**
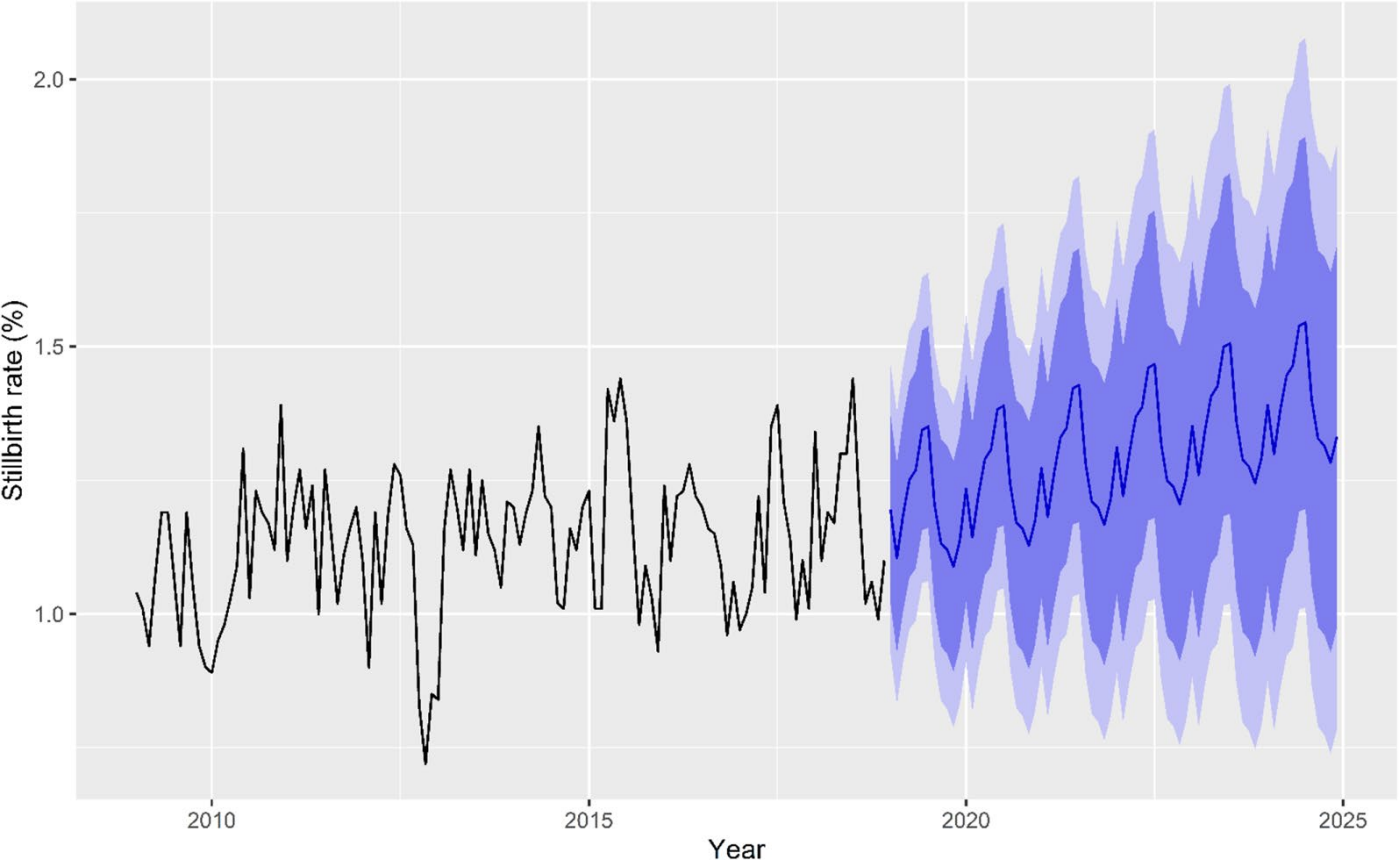
The trend plot of the forecast using the Holt-Winters exponential smoothing method. This study provided evidence of the increasing trend of stillbirth in Taiwan under global warming.

Given the season and trend effects, the data were fitted by the additive Holt-Winters seasonal exponential smoothing model. The SSE was 2.05. The Ljung-Box test showed that there was little evidence of nonzero autocorrelations in the in-sample forecast errors at lags 1–20 (chi-squared = 20.879, df = 20, *p*-value = 0.40). The distribution of forecast errors was normally distributed with a mean zero. This suggests that the autocorrelation was controlled well, and the simple exponential smoothing method can provide an adequate predictive model for stillbirth (Supplementary Fig. 2). Using the predictive model, the stillbirth rate in Taiwan is still increasing (Fig. 6).

## Discussion

This study shows that the stillbirth rate in Taiwan is on the rise. Unlike the downward trend in the global stillbirth rate from 2000 to 2019, this study shows regional differences in the stillbirth rate^4^. To the best of our limited knowledge, this is the first study to provide evidence of adverse reproductive effects of temperature on stillbirth in East Asia. This study showed an apparent harvesting effect of extreme heat on stillbirth during the third trimester of pregnancy. The harvesting effect (mortality displacement) refers to a period of excess deaths followed by a period of mortality deficit, a mortality displacement due to the death of susceptible individuals by environmental factors^6^. This study provides important evidence for the reproductive hazards caused by temperatures.

This study showed that pregnant women had an increased risk of stillbirth due to extreme heat and cold temperatures during the third trimester of pregnancy. The overall cumulative effect of hot temperatures was more significant than that of cold. Hot and cold temperatures had the most significant impacts on stillbirth during the third trimester of pregnancy, especially at about one month before delivery. These findings are similar to that of a retrospective cohort study conducted in Australia by Strand et al., in which exposure to higher ambient temperatures in the last four weeks of pregnancy increased the risk of stillbirth^7^, and a study in Texas that found evidence of an association between apparent temperature increases in the week preceding an event and the risk of stillbirth^8^. Arroyo et al.^9^conducted an ecological study in Spain that showed that exposure to the minimum temperature in the third trimester increased the risk of stillbirth. Another study in Australia by Li et al.^10^ reported that exposure to low or high temperatures in the third trimester of pregnancy significantly increased the risk of preterm birth, while the risk of stillbirth was significantly associated with exposure to low or high temperatures in the second and third trimesters of pregnancy. Identifying pregnancy windows during which women are most susceptible to adverse effects associated with exposure to extreme temperatures can determine the best periods for effective interventions. We suggest that pregnant women avoid temperature extremes, especially in the third trimester of pregnancy.

Our findings show that hot temperature has a more significant impact on stillbirth than cold temperature. The estimated risk of stillbirth in those exposed to moderate heat (25–29 °C) and extreme heat (> 29 °C) increased by 15% and 18%, respectively, compared to those exposed to the optimal temperature of 21 °C. The results were similar to those of a study in Quebec, Canada, in which exposure to an elevated outdoor temperature of 28 °C was associated with a 1.19 times higher risk of stillbirth (95% CI 1.02–1.40)^11^. Our findings are in line with earlier studies, which reported that cold seasons also increased the risk of stillbirth. An earlier study at Uppsala Hospital in Sweden from 1915 to 1929 reported that exposure to cold temperatures during pregnancy increased the risk of stillbirth^12^. A study in New York City conducted between 1940 and 1954 observed that the number of stillbirths increased in late winter and spring^13^. A survey of white singleton resident births in Minnesota from 1967 to 1973 showed that there was an increased risk of fetal death before labor in autumn and winter^14^. In Switzerland, higher rates of stillbirth were observed in winter and spring, and lower rates were observed during summer and autumn^15^. The mean stillbirth rate was 11.3 per 1000 births during our study period from 2009 to 2018. According to our forecast, the trend of stillbirths will continue to increase. As indicated in a projection of temperature-related mortality in Taiwan, the annual number of hot days with a mean temperature over 30 °C was predicted to have a substantial 2- to 5-fold increase by 2060, and heat-related mortality was predicted to increase drastically^16^. The climate has diverse effects worldwide. We suggested that additional studies in different climate zones are needed to assess the adverse effects of heat on maternal health outcomes.

There has been increasing interest in investigating the effects of heat exposure on fetal development^8^. Epidemiological studies in recent years have reported the association between extreme heat temperature, heat stress, premature birth^17^, low birth weight^18^, and stillbirth^19,20^. A systematic review of the effect of temperature on pregnancy outcomes showed stronger evidence of adverse effects due to heat than cold^3^. Current hypotheses regarding the biological mechanisms by which pregnant women are more susceptible to heat stress include (1) an increase in the core temperature due to increased fat deposition; (2) a decrease in the surface area to body mass, reducing the capacity to dissipate heat by sweating; (3) an increase in heat production due to weight gain; and (4) an increase in maternal heat stress due to the body composition and metabolic rate of the fetus^21^. The pathophysiological mechanisms might include uterine blood flow decreases under heat stress, resulting in hypoxia and increased oxidative stress, affecting placental blood flow, endocrine hormones, and metabolic functions in the placental environment, which affects fetal development^22^. We recommend that future studies investigate the biological responses and pathophysiological changes of the fetus and mother to thermal effects during pregnancy, especially in the first to the third trimester of pregnancy. However, due to ethical reasons, no studies have tested these hypotheses. We suggest that future studies develop alternative methods to observe pregnancy outcomes, such as mimicking exposure to hot environmental temperatures. For example, in a previous study, the risk of adverse birth outcomes in pregnant women who had a habit of hot tub or spa use during pregnancy was observed^23^.

The limitations of this study should be acknowledged. First, we showed that hot temperatures had a more significant overall effect on stillbirth than cold temperatures. However, Taiwan is located in a subtropical area, and the temperature is generally warm. Thus, we cannot generalize the observed effect to all countries, especially countries with cold climates. Second, we did not have information on medication, maternal infection, and congenital anomalies at an individual level. We suggest that future studies collect data on medical history and health care services, such as the number of visits, delays or non-attendance, adherence to protocol, and lack of risk assessment. Third, heat adaptation and resilience could reduce the impact of thermal hazards, but this study could not collect these effect modifiers at an individual level. We suggest that future studies collect data on adaptive behaviors, indoor temperatures, socioeconomic conditions, and cultural issues that may affect stillbirth.

In addition to direct climatic factors, thermal adaptation behaviors also indirectly affect the effects of heat stress on pregnant women^24^, such as reducing workloads during pregnancy, establishing early warning systems in the community, water supplementation, increasing medical accessibility, monitoring for heat-related conditions during labor, building modifications, and use of cooling systems^25^. We recommend more behavioral and health service-related researches to formulate recommendations for prevention.

In conclusion, we explored lag structures and performed a time-series analysis to investigate the impact of temperature on the stillbirth rate. Our study revealed that the third trimester of pregnancy is the critical exposure window, during which extreme heat and cold temperatures increased the risk of stillbirth. Pregnant women should be informed of the hazardous thermal effect on their unborn infants and protect themselves from extreme temperatures during the critical period.

## Methods

### Study design

We conducted time-series studies to investigate and forecast the impact of environmental temperature on birth outcomes in Taiwan.

#### Birth and climate data

We obtained birth data from 2009 to 2018 from the Taiwan Birth Reporting Database of the Health Promotion Administration, Ministry of Health and Welfare (https://olap.hpa.gov.tw/). It is a national registration data covering a population of 23,863,707. In Taiwan, the nationwide online birth reporting system was launched in 2004. Hospitals must report all birth certificates and birth data, including pregnancy duration, stillbirth, preterm birth, presence and type of any birth defects, and original nationality of the parturient within seven days following delivery. The database is complete and reliable due to mandatory and online entry^26^. A validation study for the birth registry in Taiwan showed a low rate of missing information and a high level of validity^27^. We obtained the monthly counts of live births and stillbirths from 2009 to 2018. In Taiwan, stillbirth is defined as the death of a fetus at more than 20 weeks of gestation or a weight of more than 500 grams^28^. The corresponding monthly mean temperature and relative humidity were obtained from the meteorological stations of the Central Weather Bureau in Taiwan^29^. The Research Ethics Committee of National Taiwan University determined whether ethical approval was required for this study (review No. 202004HM030). In Taiwan, collecting data from stillbirth cases is part of routine public health surveillance, and the available data do not contain personal identifying information. This study was therefore exempted from institutional review board assessment.

### Definition of extreme temperature

According to the World Meteorological Organization, extreme temperature is a general term for temperature variations above (extreme heat) or below (extreme cold) normal conditions. Since climate varies regionally, the definition of an extreme temperature and its threshold will differ from location to location. In other words, an extreme value in one location may be within the normal range in a different location. A simple method is to establish a specific threshold for temperature and evaluate the extremes that occur over (or under) that given threshold^30^. In this study, extreme temperatures were defined by different percentiles of T_ave_. We defined the 1st percentile (16.5 °C) and 2.5th percentile (16.7 °C) as extreme cold temperature, and 97.5th percentile (29.8 °C) and 99th percentile (30.1 °C) as extreme heat temperature.

### Statistical analysis

The analysis was performed in four steps:

#### Decomposition of time series data

To check whether there is seasonality between the stillbirth rate and temperature, we decomposed the time-series data into a trend component, a seasonal component, and an irregular component. The model was as follows:

1 $$ \begin{aligned}   ~a_{t}  &  = \alpha \left( {x_{t}  - s_{{t - \pi }} } \right) + \left( {1 - \alpha } \right)\left( {a_{{t - 1}}  + b_{{t - 1}} } \right) \\    b_{t}  &  = \beta \left( {a_{t}  - a_{{t - \pi }} } \right) + \left( {1 - \beta } \right)b_{{t - 1}}  \\    ~s_{t}  &  = \gamma \left( {x_{t}  - a_{t} } \right) + \left( {1 - \gamma } \right)s_{{t - \pi }}  \\  \end{aligned}  $$

where *a*_*t*_ is the horizontal component of the sequence, *b*_*t*_ is the trend component of the sequence, and *s*_*t*_ is the seasonal factor of the sequence (π is the cycle length of the sequence)^31^.

#### Estimate the burden of stillbirth attributable to non-optimal temperature

Because the distribution of the number of stillbirths follows a Poisson distribution, we conducted Poisson regression to calculate the RR of stillbirth and estimate the burdens of cold and heat-related stillbirths. We estimated the burdens of heat-related stillbirths and cold-related stillbirths by calculating the AF of stillbirths using the following equation:

2 $$ {\text{AF}} = \frac{{{\text{(RR}} - {1)}}}{{{\text{RR}}}}{{ \times }}100 $$

where RR is the relative risk of cold or hot temperatures considering the optimal temperature as the reference temperatures, which corresponded to the time points with the minimum stillbirth rates. We calculated the number of stillbirths attributable to exposure to hot and cold temperatures, defined as temperatures above (> 29 °C) or below (< 20 °C) the optimal temperature, respectively, which corresponded to the minimum stillbirth rate. The total number of stillbirths attributable to non-optimal temperature exposure was calculated as the sum of the contributions from cold and hot temperatures; the total attributable fraction was defined as the ratio of the summed value to the total number of stillbirths.

#### Lag and nonlinear effect analysis

Because the association between temperature and stillbirth might be non-linear, and there might be a lag effect of temperature that may affect stillbirth on given periods after exposure. We applied generalized linear quasi-Poisson regression combined with the DLNM to identify the delayed effects of ambient temperature on the stillbirth rate^32^. The model was as follows:

3 $$  {\text{Log}}\left[ {E\left( {Y_{t} } \right)} \right]{\text{ }} = {\text{ }}\alpha {\text{ }} + {\text{ }}\beta \,Temp_{{t,l}} ~ + NS\left( {{\text{RH}}_{t} ,{\text{ df}}} \right)~ + NS\left( {{\text{year}},{\text{ df}}} \right){\text{ }} + NS\left( {{\text{month}},{\text{ df}}} \right)  $$

where *t* is the month of observation; *E(Y*_*t*_*)* is the expected number of stillbirths in month *t*; β is the vector of coefficients for *Temp*_*t,l*_; *Temp*_*t*_,_*l*_ is a matrix representing the two-dimensional relationship of mean temperature and lag months; *l* denotes the maximum lag months; RH_t_ is the relative humidity (RH) in the *t* months of observation; *NS*() represents the natural cubic spline. Considering that the impacts of temperature on stillbirth may occur with delay, cross-basis *Temp*_*t,l*_ was constructed, with a quadratic B-spline with three internal knots placed at equally spaced values in the log scale lags^33,34^. To estimate the effect of temperature on all stages of pregnancy, we specified a maximum lag of 9 months. Degrees of freedom (df) for the lag structure was selected based on the seasonal pattern and trend observed from the decomposition of the time-series analysis and Akaike information criterion (AIC)^35^. We used a natural cubic spline with 10 dfs per year to adjust for the long-term trend during human gestation, and a natural cubic spline with 12 dfs per month to adjust for the monthly variation^34^. The final model used a quadratic B-spline with 5 dfs and a second-order quadratic B-spline with 4 dfs. A natural cubic spline with 3 dfs was applied for the relative humidity^36^. We identified the temperature corresponding to the lowest risk as to the optimal temperature and the reference value. Then, we obtained estimates for the heat effect on stillbirth^37^. To estimate the effects of extreme temperature on stillbirth, we defined the 99th and 97.5th percentiles of temperature as extremely high temperatures and the 1st and 2.5th percentiles as extremely low temperatures to calculate the risk relative to the reference value, which corresponded to the lowest stillbirth rate. We also calculated the number and fraction of stillbirths attributable to extreme temperatures, defined using cutoffs at the 2.5th and 97.5th temperature percentiles and the estimated exposure-lag-response association defined by the DLNM^37^.

#### Projection of stillbirth rate

We constructed the additive Holt-Winters exponential smoothing model to forecast the stillbirth rate until 2025^38^. Temporal autocorrelation is common in time-series analyses, in which consecutive outcome data may be highly correlated. To test whether there were nonzero autocorrelations in the in-sample forecast errors, we performed the Ljung-Box test^39^. To ensure that the predictive model was optimized, we assessed whether the forecast errors were normally distributed with mean zero and constant variance. We measured the accuracy of the predictive model using the sum of squared error (SSE) to assess in-sample forecast errors.

We used the "forecast" package of R software for time-series forecasting^40^ and the "dlnm" package for distributed lag nonlinear modeling^41^. A *P*-value less than 0.05 was considered to be statistically significant.

## Supplementary Information

Supplementary Information.

## Data Availability

The data that support the findings of this study are available upon request from the corresponding author (H.-Y.Y.).
