# Supplementary material for: Extreme temperature increases the risk of stillbirth in the third trimester of pregnancy
Source: Sci Rep. 2022 Nov 2;12:18474. doi: 10.1038/s41598-022-23155-3 (PMC9630541; doi:10.1038/s41598-022-23155-3)
Supplement: Supplementary file 1 — Supplementary Information. [file 41598_2022_23155_MOESM1_ESM.pdf]

Title: Extreme temperature increases the risk of stillbirth in the third trimester of pregnancy

Author names: Hsiao-Yu Yang[1,2,3]*, Jason Kai Wei Lee[4,5,6,7,8,9], Chia-Pin Chio[1,10]

Affiliations:

[1] Institute of Environmental and Occupational Health Sciences, National Taiwan University College of Public Health, Taipei, Taiwan

[2] Department of Public Health, National Taiwan University College of Public Health, Taipei, Taiwan

[3] Department of Environmental and Occupational Medicine, National Taiwan University Hospital, Taipei, Taiwan

[4] Human Potential Translational Research Programme, Yong Loo Lin School of Medicine, National University of Singapore, Singapore

[5] Department of Physiology, Yong Loo Lin School of Medicine, National University of Singapore, Singapore

[6] Global Asia Institute, National University of Singapore, Singapore

[7] N.1 Institute for Health, National University of Singapore, Singapore

[8] Institute for Digital Medicine, National University of Singapore, Singapore

[9] Singapore Institute for Clinical Sciences, Agency for Science, Technology, and Research (A*STAR),

Singapore

[10] Department of Medical Research, Tung' Taichung Metro Harbor Hospital, Taichung, Taiwan

* Corresponding author: Dr. Hsiao-Yu Yang hyang@ntu.edu.tw

Address: No. 17 Xuzhou Road, Taipei 10055, Taiwan

Supplementary Table 1. Comparison of temperature and stillbirth in each month in Taiwan, 2009—2018.

Month	Mean temperature	Relative humidity (%)	Number of births	Number of stillbirths	Stillbirth rate (%)
Jan	17.2±1.1	75±5	173,553	1,900	1.10±0.16
Feb	18.0±1.4	77±3	153,394	1,616	1.06±0.10
Mar	19.8±1.1	75±3	165,160	1,870	1.13±0.12
Apr	23.1±1.0	75±3	156,094	1,837	1.18±0.12
May	26.3±0.8	76±4	149,563	1,943	1.22±0.11
Jun	28.4±0.8	77±2	157,712	1,983	1.26±0.12
Jul	29.5±0.4	74±1	166,655	2,061	1.23±0.14
Aug	29.1±0.6	76±2	172,886	1,980	1.15±0.10
Sep	28.2±0.7	74±2	173,082	1,897	1.10±0.08
Oct	25.3±0.8	74±3	188,174	1,992	1.07±0.10
Nov	22.8±0.8	76±2	177,929	1,800	1.02±0.13
Dec	18.7±1.2	74±3	175,455	1,886	1.09±0.17
**Total**	**23.9±4.5**	**75±3**	**2,019,557**	**22,769**	**1.13±0.14**

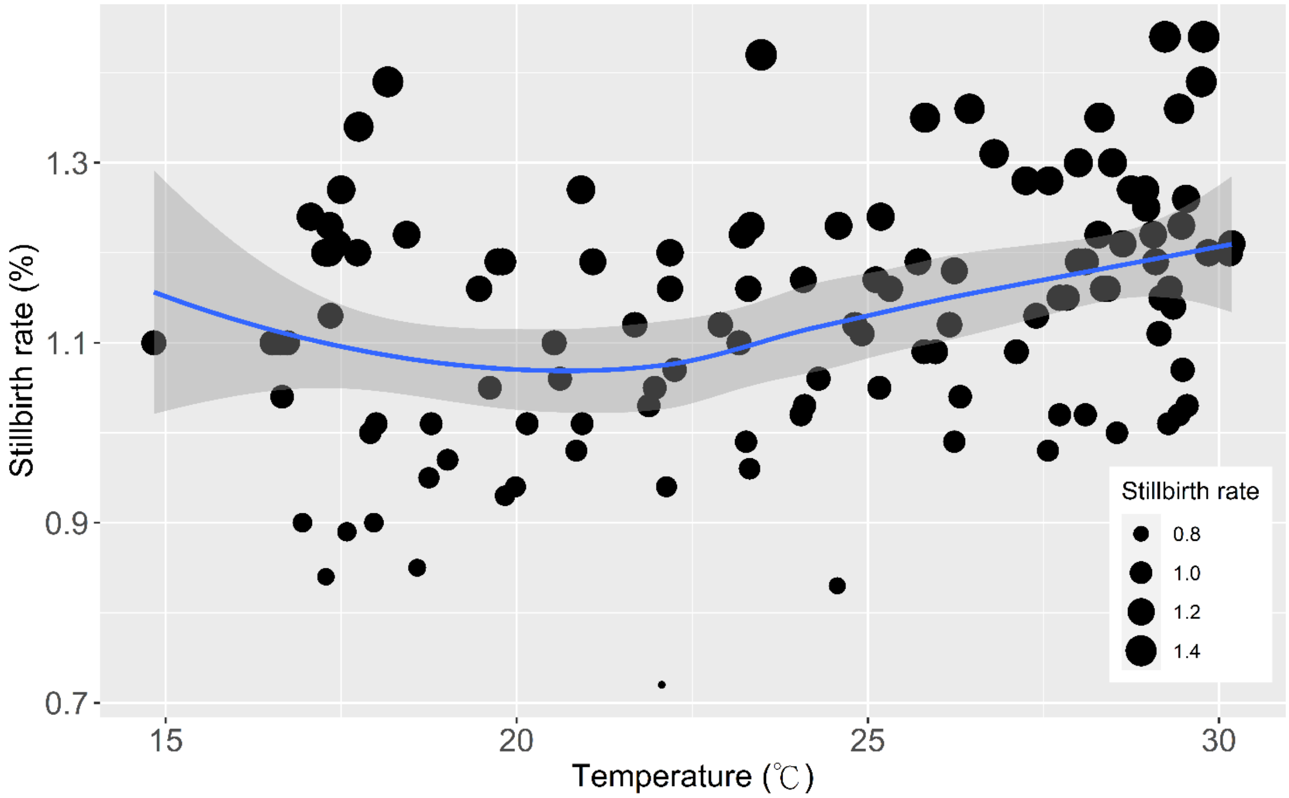

Supplementary Fig. 1. Scatter plot and nonlinear regression line of temperatures and stillbirths.

Legend: There was a nonlinear association between temperature and the stillbirth rate. The minimum stillbirth rates occurred at temperatures between 20°C and 22°C.

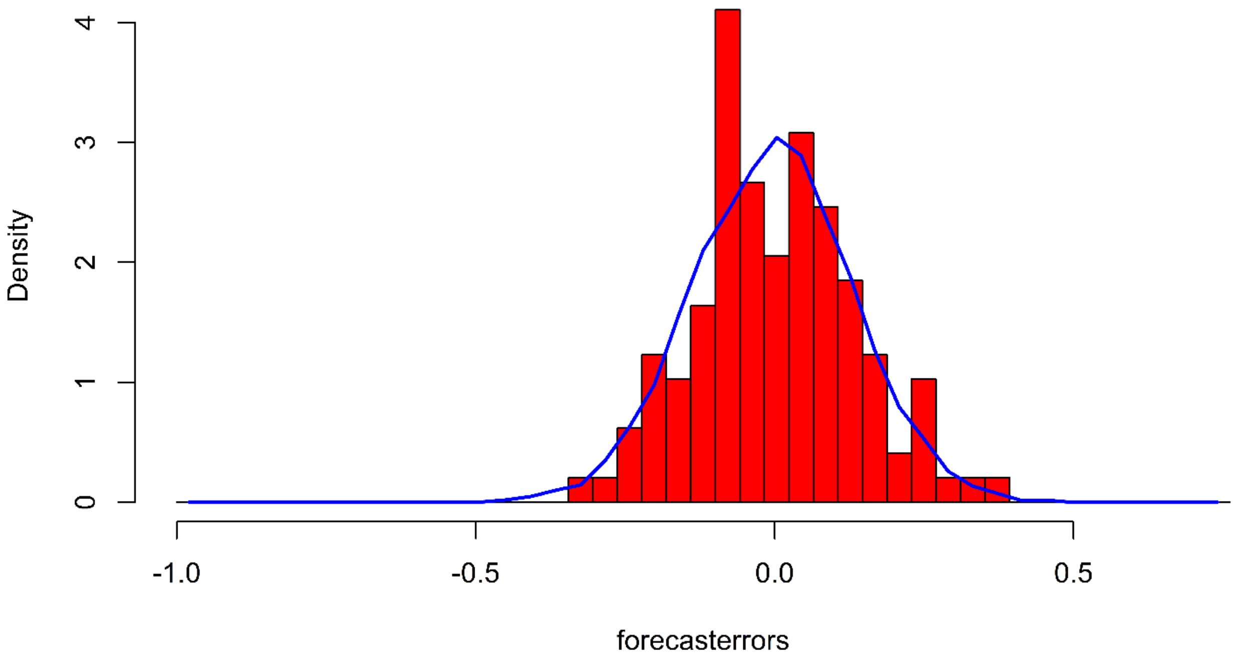

Supplementary Fig. 2. Histogram of forecast errors.

Legend: The plot shows that the distribution of forecast errors is roughly centered on zero and is more or less normally distributed.
